# Neural Dynamics of Audiovisual Synchrony and Asynchrony Perception in 6-Month-Old Infants

**DOI:** 10.3389/fpsyg.2013.00002

**Published:** 2013-01-21

**Authors:** Franziska Kopp, Claudia Dietrich

**Affiliations:** ^1^Center for Lifespan Psychology, Max Planck Institute for Human DevelopmentBerlin, Germany

**Keywords:** multisensory perception, audiovisual, infancy, ERP, synchrony, asynchrony

## Abstract

Young infants are sensitive to multisensory temporal synchrony relations, but the neural dynamics of temporal interactions between vision and audition in infancy are not well understood. We investigated audiovisual synchrony and asynchrony perception in 6-month-old infants using event-related brain potentials (ERP). In a prior behavioral experiment (*n* = 45), infants were habituated to an audiovisual synchronous stimulus and tested for recovery of interest by presenting an asynchronous test stimulus in which the visual stream was delayed with respect to the auditory stream by 400 ms. Infants who behaviorally discriminated the change in temporal alignment were included in further analyses. In the EEG experiment (final sample: *n* = 15), synchronous and asynchronous stimuli (visual delay of 400 ms) were presented in random order. Results show latency shifts in the auditory ERP components N1 and P2 as well as the infant ERP component Nc. Latencies in the asynchronous condition were significantly longer than in the synchronous condition. After video onset but preceding the auditory onset, amplitude modulations propagating from posterior to anterior sites and related to the Pb component of infants’ ERP were observed. Results suggest temporal interactions between the two modalities. Specifically, they point to the significance of anticipatory visual motion for auditory processing, and indicate young infants’ predictive capacities for audiovisual temporal synchrony relations.

## Introduction

In natural environments, continuous streams of multisensory stimuli are processed in the human brain. When these stimuli occur at the same spatial location and in temporal synchrony, perception as unitary events originating from the same source is very likely (Welch and Warren, 1980). Temporal synchrony seems to be a particularly strong binding cue (e.g., Spence and Squire, 2003). For audition and vision, neural activity in large-scale networks has been found to interact with synchrony relations (e.g., Bushara et al., 2001; Macaluso et al., 2004; Miller and D’Esposito, 2005). Empirical evidence suggests that, within temporal proximity, stimuli from the two sensory modalities are pulled into temporal alignment and perceived as unitary events (e.g., Lewkowicz, 1996; Fendrich and Corballis, 2001). The dynamics by which the cognitive system creates synchronous versus asynchronous percepts have a certain degree of flexibility, depending on a variety of factors such as stimulus complexity, familiarity and experience, and repeated asynchrony presentation (e.g., Dixon and Spitz, 1980; Fujisaki et al., 2004; Vatakis et al., 2007; Petrini et al., 2009a). Importantly, these temporal binding mechanisms appear to undergo changes across the lifespan (Lewkowicz, 1996, 2010; Hay-McCutcheon et al., 2009).

Research in animals and humans suggests that multisensory interactions develop gradually during postnatal life, and that early experience is critical for the maturation of multisensory capabilities (e.g., Wallace et al., 2004; Putzar et al., 2007). Human infants are sensitive to temporal synchrony relations of auditory and visual events from early on. For example, Dodd (1979) demonstrated that 10- to 16-week-old infants looked longer at an experimenter reciting nursery rhymes when the lip movements and the speech sounds were presented with an asynchrony of 400 ms as compared to synchrony. But this ability is not limited to speech stimuli. In a habituation paradigm, infants as young as 4 weeks detected a change in audiovisual synchrony relations in naturalistic objects striking against a surface (Bahrick, 2001). Similarly, in a visual-preference task, 4-month-old infants preferred synchronous over asynchronous presentations of moving stuffed animals with the corresponding impact sounds (Spelke, 1979). Moreover, a number of behavioral studies have demonstrated that, starting from birth (Lewkowicz et al., 2010), human infants rely on audiovisual synchrony for intersensory matching (e.g., Bahrick, 1983; Lewkowicz, 1986, 1992, 2000; Lewkowicz et al., 2008). However, little is known about how the infant neural system creates a synchronous or asynchronous percept. Lewkowicz investigated the temporal window in which auditory and visual stimuli are bound together to a unitary event and found that infants in the first year of life are less sensitive to audiovisual asynchrony than adults. For instance, infants tolerate temporal disparities of auditory signals preceding visual events by about 300 ms in bouncing, sounding objects (Lewkowicz, 1996) and by 500 ms or more in speech stimuli (Lewkowicz, 2010) and still perceive simultaneity. Apparently, the greater asynchrony tolerance does not change between 2 and 10 months.

Much of the empirical evidence for infant responses to multisensory stimuli stems from behavioral research paradigms, such as visual paired-preference tasks or habituation-test procedures. However, little is known about the neural mechanisms underlying synchronous and asynchronous multisensory perception in the infant brain. Insight into the emergence of such percepts could help to take an infant perspective and to understand multisensory processes in the less experienced perceptual system. As an adequate method, non-invasive EEG measures have been useful in exploring the dynamics of perceptual and cognitive processes early in development. Here we assessed event-related brain potentials (ERP) that allow tracking of neural signatures with high temporal resolution and reveal dynamics of the neural activity underlying audiovisual temporal binding processes.

In human adults, ERPs were found to be modulated by multisensory interactions. In particular, the auditory components N1 and P2 were sensitive to audiovisual presentations (for reviews, see Fort and Giard, 2004; Besle et al., 2009). N1 and P2 amplitude suppressions have been observed in bimodal as compared to unimodal events (e.g., Besle et al., 2004; van Wassenhove et al., 2005; Stekelenburg and Vroomen, 2007). N1 and P2 latencies were shortened with audiovisual presentations, indicating faster processing in congruent multisensory events (van Wassenhove et al., 2005; Stekelenburg and Vroomen, 2007; Vroomen and Stekelenburg, 2009). ERP responses were also found to vary with temporal synchrony relations. Pilling (2009) replicated N1 and P2 amplitude decreases in synchronous audiovisual speech stimuli compared to unisensory auditory stimuli, but did not observe such amplitude suppressions when the auditory onset occurred 200 ms earlier. Similarly, Vroomen and Stekelenburg (2009) found that N1 and P2 amplitude modulations varied with the introduction of temporal asynchronies in audiovisual events. Importantly, some N1 and P2 modulations in multisensory events could only be observed when the stimuli contained salient visual inputs (van Wassenhove et al., 2005) or anticipatory visual motion, thus making the auditory onset predictable (Stekelenburg and Vroomen, 2007; Vroomen and Stekelenburg, 2009).

ERP research on multisensory perception early in ontogeny is still scarce. Hyde et al. (2010) investigated 3-month-olds’ neural responses to bimodal versus unimodal presentation of circles and tones. In contrast to a number of findings in adults (see above), early auditory components were not sensitive to the experimental manipulation at this age. Rather, ERP responses differentiated between multisensory and unisensory presentations later in the analysis epoch (between 400 and 550 ms). However, P2 mean amplitude modulations were observed in 5-month-olds (Hyde et al., 2011), suggesting increasing responsiveness of this auditory ERP component in the more mature perceptual system. In their study, Hyde et al. (2011) found greater P2 amplitudes for audiovisual synchronous stimuli including a speech sound and a static face as compared to asynchronous stimuli including the same speech sound with the static face onset delayed by 400 ms. In a second experiment, larger P2 amplitudes were found for dynamic synchronous speech stimuli as compared to stimuli in which the facial movements in the visual stream did not correspond to the audio stream (Hyde et al., 2011). These findings demonstrate ERP modulations as a response to differences in multisensory perception in young infants. However, based on this research, conclusions about the processing of audiovisual temporal synchrony relations are difficult, because synchrony and asynchrony were confounded with static versus dynamic presentation, identity change, and manipulation of the visual onset.

In the present study, we aimed at exploring neural mechanisms of real audiovisual synchrony versus asynchrony perception in infancy by presenting dynamic video and sound stimuli. Thus, we avoided possible confounds with changes in static versus dynamic stimulus presentation (see Hyde et al., 2011). We applied a standardized infant-controlled habituation-test procedure to validate the individuals’ capacities to detect a 400-ms asynchrony (see Lewkowicz, 1996) and assessed EEG responses to ecologically valid, audiovisual events (Stekelenburg and Vroomen, 2007) including socially relevant, communicative signals (De Gelder and Bertelson, 2003; Vatakis and Spence, 2006). We refrained from presenting speech stimuli to prevent interferences with individual language development processes in the preverbal infants. Instead, we produced videos of a person clapping her hands (Stekelenburg and Vroomen, 2007). Audiovisual asynchrony was achieved by delaying the content of the visual stream by 400 ms (see Materials and Methods and Figure 2) while keeping both the video and audio onset times and durations identical between the synchrony and asynchrony condition (cf. Doesburg et al., 2007). In contrast to several previous research paradigms (Pilling, 2009; Vroomen and Stekelenburg, 2009; Hyde et al., 2011), this setup avoided differences due to attentional shifts as orienting responses to stimulus onsets or offsets during the presentation, and attentional competition between the two sensory modalities (Talsma et al., 2010). Moreover, this design made it possible to keep the content of the stimulus constant, thereby avoiding a possible confound with change of congruency between the auditory and visual streams (e.g., Hyde et al., 2011). The magnitude of the audiovisual delay was piloted using a habituation paradigm. Earlier data have confirmed young infants’ responses to a 400 ms audiovisual asynchrony in non-speech stimuli (Lewkowicz, 1996). The participants in the present study were 6-month-old infants. This age group was selected for several reasons. First, previous behavioral data have shown sensitivity to multisensory temporal relations in even younger infants (e.g., Bahrick, 2001; Lewkowicz et al., 2010). In other words, 6-month-olds are assumed to be able to detect the asynchrony. Second, it appears that the size of the temporal range of audiovisual integration does not change reliably in the first year of life (Lewkowicz, 1996). Third, as noted above, the general sensitivity of some early ERP components to the manipulation of multisensory phenomena seems to be more pronounced in infants older than 3 months (Hyde et al., 2010, 2011). Finally, 6-month-olds are awake and attentive enough to conduct an EEG experiment over a period of several minutes that yields sufficient data for ERP averaging. At the same time, the probability of spontaneous or even coordinated imitative behavior as a response to the hand-clapping stimuli is still very low at this age.

It is known that infant and adult ERP components may differ substantially in terms of amplitude, latency, or polarity (de Haan, 2007). Intra- and inter-individual variability is high in the immature ERP (de Haan, 2007), with variability in neural responses decreasing during the first months of life (Thomas et al., 1997). Despite these differences as compared to the adult ERP, the auditory components N1 and P2 have also been identified in infants. Wunderlich et al. (2006) reported (a) significantly longer N1 and P2 latencies in infants than in adults, (b) increases of N1 and P2 amplitude with age, and (c) more uniform scalp distributions in young infants and toddlers, but more focal distributions in older children and adults. For the present study, we expected similar results as those found in human adults during multisensory perception, that is, amplitude and/or latency modulations of the auditory components N1 and P2 (Stekelenburg and Vroomen, 2007; Pilling, 2009; Vroomen and Stekelenburg, 2009).

Regarding the multisensory nature of the stimuli, infant-specific ERP components related to visual recognition processes are relevant for this experiment. The most pronounced component is Nc, a large negative deflection with a fronto-central distribution, peaking between 400 and 700 ms after stimulus onset. This component has been shown to relate to attentional processing, to orientation to salient stimuli, and to memory, as it was correlated with stimulus novelty (e.g., de Haan and Nelson, 1997; Ackles, 2008; Kopp and Lindenberger, 2011, 2012). A second, less well understood infant ERP component is Pb, a positive deflection peaking between 250 and 450 ms. It has been associated with processes of stimulus expectancy (e.g., Karrer and Monti, 1995) and with mechanisms related to the relevance of stimuli, particularly as coded over long-term retention periods (Kopp and Lindenberger, 2011, 2012). It is conceivable that neural mechanisms related to attention, as reflected in Nc, and neural mechanisms related to expectancy, as reflected in Pb, are both modulated by audiovisual synchrony versus asynchrony perception.

## Materials and Methods

### Participants

A total of 47 6-month-old infants were tested. All infants were born full-term (≥38th week), with birth weights of 2500 g or more. According to the respective caregiver’s evaluation, all participants were free of neurological diseases, and had normal hearing and vision. In the behavioral habituation experiment, two infants were excluded due to an experimental error (*n* = 1) or failure to fulfill the fatigue criterion (see below; *n* = 1). The final sample consisted of 45 infants (28 girls, 17 boys) with an age range of 170–195 days (*M* = 178.8 days, SD = 6.2 days).

Thirty infants who had been tested in the habituation experiment were not included in the final EEG analysis due to insufficient behavioral asynchrony discrimination in the habituation experiment (see below; *n* = 13), or failure to reach the minimum requirements for adequate ERP averaging (*n* = 17), for example, because of excessive fussiness, movement artifacts, or insufficient visual fixation. The final sample of the EEG experiment included 15 infants (10 girls, 5 boys) with an age range between 171 and 193 days (*M* = 177.3 days, SD = 5.5 days). The Ethics Committee of the Max Planck Institute for Human Development, Berlin approved this study. Informed written consent was obtained from the infants’ caregivers.

### Habituation experiment

#### Stimuli

**Figure 1:**
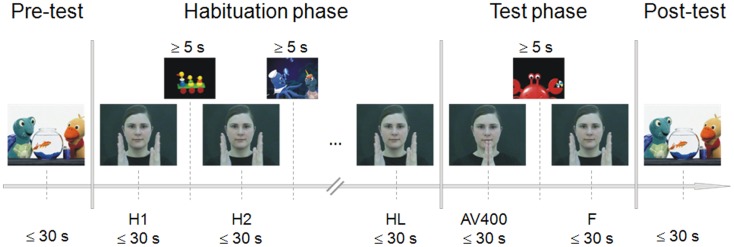
**Experimental procedure of the habituation paradigm**. During pre- and post-test, a sequence of a child movie was presented to control for fatigue effects. Habituation was reached by repeated presentation of the audiovisual synchronous stimulus (H1, H2, …). After the last habituation trial (HL), the novel asynchronous stimulus (AV400) was shown, followed again by the presentation of the familiar synchronous stimulus (F). Each experimental stimulus was presented as long as the infant looked at it or for a maximum duration of 30 s. To attract the child’s attention back to the screen, further sequences of the child movie were presented between habituation trials and between test trials. The child had to look at the screen for at least 5 s in order to continue with stimulus presentation.

**Figure 2:**
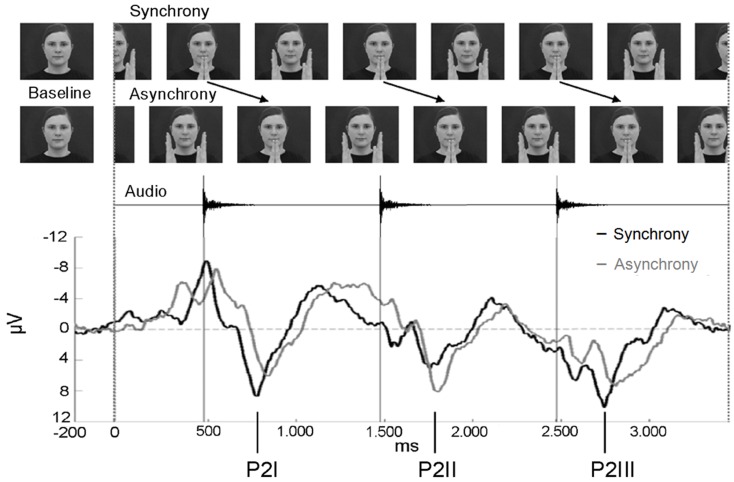
**Stimulus presentation of the EEG experiment and ERP epoch between −200 and 3400 ms**. In asynchronous trials, the visual stream was delayed by 400 ms to the auditory stream. The ERPs of the synchrony (black line) and the asynchrony condition (gray line) are averaged across anterior electrodes for the purpose of illustration (*n* = 15). Note that the auditory onset was at the same point in time in both conditions. The three P2 components elicited during the entire epoch are labeled P2I, P2II, and P2III.

Videos were produced showing a female person who was highly trained in clapping her hands in a rhythm with equidistant intervals of 1000 ms. She was instructed to synchronize her movements to audio signals presented to her left ear via a hidden headphone. A camera captured her face down to her shoulders, together with her hand movements in front of her face (see Figures 1 and 2), at a rate of 25 frames per second. Her facial expression was neutral with a slight smile to appear friendly to the child.

The parts of the footage that were most similar with respect to volume and precision of the clapping interval were chosen for further editing. Inter-clapping intervals deviated from 1000 ms by ±21.7 ms on average. A synchronous and an asynchronous stimulus were created, each with a duration of 30 s. In the asynchronous hand-clapping sequence, the visual stream was delayed with respect to the sound by 400 ms (10 frames). A 30-s sequence of an age-appropriate child movie was presented at the beginning and at the end of the habituation experiment to control for signs of fatigue. Loudness of both the audiovisual clapping sequences and the child movie sequences was 53 dB SPL (A).

#### Procedure

Infants were tested in a quiet, acoustically shielded room, in an area surrounded by white curtains to prevent visual distraction. The infant sat on the caregiver’s lap. A computer screen was placed in front of the infant (distance 80 cm), and two loudspeakers were placed to the left and to the right of the screen with a distance of approximately 100 cm to the infant’s head. The infants’ behavior was videorecorded to verify gaze durations during the experiment and again later off-line.

The procedure of the infant-controlled habituation paradigm was adapted from Lewkowicz (1996, 2010) and Flom and Bahrick (2007). The paradigm included a pre-test, habituation phase, test phase, and post-test (Figure 1). Pre- and post-tests served as controls for alertness before habituation trials and after test trials. For this purpose, sequences of the child movie were presented for as long as the child looked at them or for a maximum duration of 30 s. Looking times of less than 5 s in the pre- and/or post-test led to the exclusion of the infant (fatigue criterion). The movie sequences were the same for pre- and post-test.

The habituation phase included repeated presentations of the synchronous clapping stimulus. Each of the habituation trials lasted as long as the child looked at the screen or for a maximum duration of 30 s. An experimenter monitored the infant’s gazing behavior. Whenever the infant looked away from the screen, she pressed a button and another sequence of the child movie appeared. The gaze away from the monitor had to last at least 1 s including a head movement. The child movie sequences between the habituation trials served to attract attention, and when the infant looked at them for at least 5 s, the experimenter switched to the next habituation trial. The habituation criterion was reached when the mean gaze duration to the last three habituation trials was shorter than 50% of the mean gaze duration to the first three habituation trials. Thus, the minimum number of habituation trials was six.

In the test phase, the familiar synchronous stimulus (F) and the novel asynchronous test stimulus (AV400) were presented to test for recovery of interest. Again, the experimenter pressed a button when the child no longer looked at the screen or after a maximum of 30 s. Sequences of the child movie appeared to attract the infant’s attention, and when the child looked at the monitor for at least 5 s, the experimenter switched again to the next test trial. This habituation-test procedure was applied to estimate individually whether or not the infant was able to discriminate the AV asynchrony of 400 ms. Therefore, the order of test trial presentation was always the same across children. Infants saw and heard the last habituation trial (HL), then AV400, and then F before the post-test trial was presented.

#### Data analysis

An asynchrony discrimination score (ADS) based on individual gaze durations was calculated by dividing the looking time to AV400 by the mean value of looking times to HL and F^1^. Infants with an ADS lower than 1.2 were not included in further EEG assessment. This criterion was chosen based on prior pilot data in our lab. It is rather strict considering that discrimination effects are usually revealed in group-level data. However, we aimed to establish that the individual had shown recovery of interest to the asynchronous test trial.

### EEG experiment

#### Stimuli

Two stimuli were created by cutting a sequence from the videos in which the female clapped her hands rhythmically at a rate of precisely 1000 ms (Figure 2). The synchronous stimulus consisted of four hand-clapping movements in synchrony with the corresponding hand-clapping sound. For the final analysis, EEG epochs comprised the first 3400 ms after stimulus onset with sounds occurring at 474, 1474, and 2474 ms. The visual stimulus occupied a visual angle of 8.7° when the female had extended both arms, and 7.1° when her palms had clapped. The four hand-clapping sounds had an equalized sound pressure level of 48 dB SPL (A), each with a rise time of 10 ms and a fade-out period of 170 ms.

The asynchronous stimulus was generated by including a visual delay of 400 ms. That is, the video stream of the final EEG epochs showed the hands of the female clapping at 874, 1874, and 2874 ms after stimulus onset. A 400-ms video sequence of the continuous movement was added at the beginning and the video sequence of 400 ms at the end was cut out. In contrast, the time course of the auditory stream did not differ from the synchronous stimulus in terms of sound onset (at 474, 1474, and 2474 ms after video onset).

#### Procedure

The EEG experiment was conducted during the same test session. Right after the habituation paradigm, EEG equipment was set up. Surroundings, experimental setting, and video recording were the same as in the habituation experiment. Caregivers were briefed not to talk to the child or to point at the screen, nor to interact in any way with the child, and to avoid any movements to minimize EEG artifacts.

Synchronous and asynchronous stimuli were presented in random order in an event-related design. First, each trial started with an alternating sequence of an animated child movie to direct and maintain the child’s attention to the screen. These animated movie sequences were randomly selected for each trial out of 20 sequences with varying durations between 3000 and 6000 ms (in steps of 500 ms). Second, in both experimental conditions, a static photo of the female was presented for 1000 ms as a baseline. In order to make the transition between baseline and stimulus as smooth as possible, a snapshot of the subsequent clapping stimulus was adapted to create the baseline photo. The hands of the woman were removed and replaced by the background of the picture (Figure 2). For data analysis, the last 200 ms of the static image presentation were used as the pre-stimulus baseline for the ERP epochs. Third, the synchronous or the asynchronous clapping stimulus was presented for 4000 ms. No more than three synchronous or three asynchronous trials were presented consecutively. Whenever the infant became fussy or did not look at the screen any longer, an age-appropriate animated movie was presented. When the infant attended to the screen again, the presentation of stimuli continued. The session ended when the infant’s attention could no longer be attracted to the screen. Within the session, a maximum of 90 trials of synchronous and 90 trials of asynchronous stimuli were presented. Infants saw and heard on average 35.2 synchronous (SD = 7.6) and 33.1 asynchronous stimuli (SD = 5.5).

#### EEG acquisition and analysis

EEG signals were continuously recorded at 32 active electrodes with a sampling rate of 1000 Hz and amplified by a Brain Vision amplifier. The reference electrode was placed at the right mastoid, and the ground electrode at AFz. Signals at FP1 and FP2 were monitored to check for vertical eye movements, and signals at F9 and F10 were checked for horizontal eye movements. Impedances were kept below 20 kΩ.

All trials in which the infant did not look at the screen were excluded from further analysis. EEG was re-referenced off-line to linked mastoids (Junghöfer et al., 1999). A bandpass filter was set off-line between 0.5 and 20 Hz. ERP epochs comprised the 200-ms baseline before video onset followed by 3400 ms of video presentation. Artifacts due to eye or body movements or external sources were automatically discarded when voltage exceeded ±120 μV. In addition, EEG signals were inspected visually to scan for and reject artifacts. A baseline correction to the 200-ms pre-stimulus baseline was performed. Finally, individual averages (ERP) and grand averages across subjects were calculated. For ERP analysis, infants contributed an average of 24.0 trials with synchronous (SD = 7.9), and 23.0 trials with asynchronous stimuli (SD = 7.4) to grand averages.

## Results

### Behavioral data

**Figure 3:**
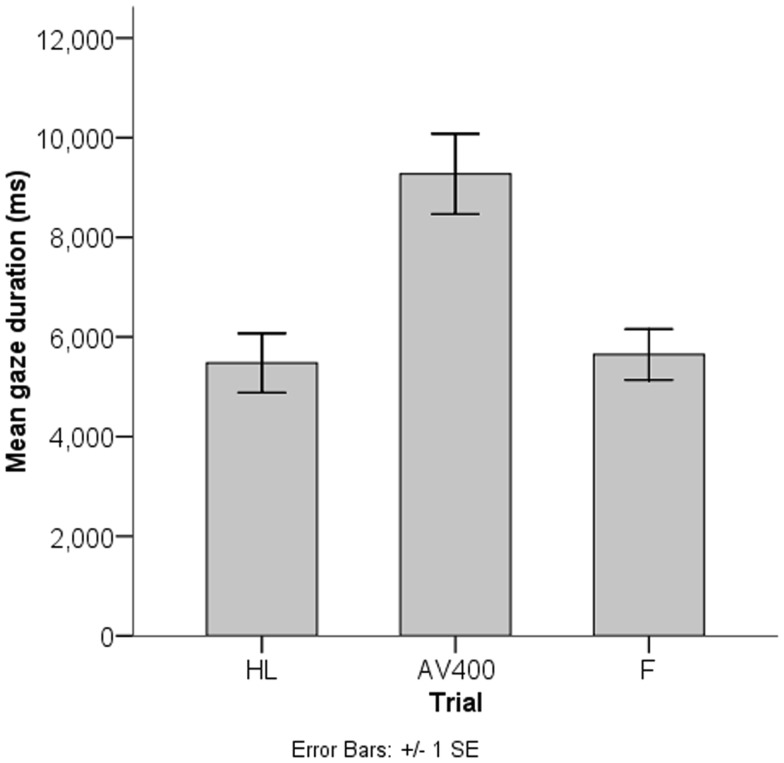
**Results of the behavioral habituation paradigm**. Infants (*n* = 45) looked to the asynchronous test stimulus (AV400) for longer on average than to the familiar synchronous stimulus as shown for the last habituation trial (HL) and the familiar test trial (F).

Figure 3 shows mean gaze durations of the habituation-test paradigm. Infants reached habituation to the synchronous trial on average after 9.5 presentations. Average gaze durations to the animated child movie in the pre-test (*M* = 27115.8 ms, SD = 5406.8 ms) and post-test (*M* = 23821.3 ms, SD = 7503.8 ms) were long, indicating high alertness during the whole test procedure.

The mean looking time to AV400 (*M* = 9272.8 ms, SD = 5416.2 ms) was reliably longer than to F (*M* = 5646.7 ms, SD = 3416.1 ms), *t*(44) = 4.26, *p* < 0.001. This result indicates that, on the group-level, infants showed recovery of interest after habituation to the synchronous stimulus and discriminated the AV asynchrony of 400 ms. In line with the calculation of the ADS, an additional paired-samples *t* test between gaze durations to AV400 and the mean of the gaze durations to HL and F (*M* = 5563.1 ms, SD = 2875.1 ms) revealed a similar result, *t*(44) = 5.30, *p* < 0.001. That is, discrimination of the AV asynchrony of 400 ms could be established.

On average, ADS were 1.8 (SD = 1.0, Min = 0.6, Max = 5.5) in this sample (*n* = 45). As explained above, infants with an ADS lower than 1.2 were excluded from the ERP analysis. Thirty-two infants (71.1%) showed adequate AV asynchrony discrimination with a mean ADS of 2.2 (SD = 1.0, Min = 1.2, Max = 5.5). A possible sampling bias with respect to sensitivity to audiovisual temporal synchrony relations cannot be fully excluded for the EEG experiment. However, non-significant visual recovery of interest to the AV400 trial after habituation to the synchronous stimulus cannot necessarily be referred to as a lack of discrimination of the asynchrony. Therefore, analyzing the neural activity patterns in infants who did not meet the ADS criterion seems preferable. Only *n* = 3 out of those *n* = 13 children provided EEG data sufficient for ERP analysis. Thus a meaningful statistical analysis was not possible.

### EEG data

#### ERP components and overview of analysis

**Figure 4:**
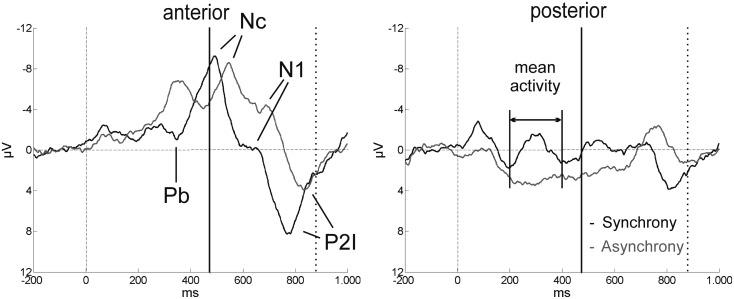
**ERP components in the first 1000 ms after video onset**. The ERPs for synchrony (black line) and asynchrony (gray line) are averaged across all anterior and all posterior electrodes for the purpose of illustration (*n* = 15). Solid vertical lines at 474 ms indicate the auditory onset in both experimental conditions. The visual clapping of the hands occurred at 874 ms in the asynchronous condition (indicated by dotted vertical lines).

The most salient component was elicited as a response to the presentation of the auditory event (onsets at 474, 1474, and 2474 ms, respectively) and may correspond to the adult P2. This component was analyzed for all three sounds within the 3400 ms epoch and termed P2I (interval: 700–900 ms after video onset), P2II (interval: 1700–1900 ms after video onset), and P2III (interval: 2700–2900 ms after video onset), respectively (Figure 2). P2 peaks occurred on average 284.7 ms after sound onset in synchronous stimuli and on average 346.0 ms in asynchronous stimuli. Prior to P2I, an auditory N1 was elicited (interval: 550–750 ms after video onset). The N1 activity partly overlapped with responses to the visual stimulation before the auditory onset (see Figure 4), however, the N1 peak could be identified and was therefore analyzed. N1 peaked on average at 156.3 ms after sound onset in synchronous stimuli and at 220.8 ms in asynchronous stimuli. In the time course of the epoch, ERP activity seemed to smear progressively (Figure 2). Therefore, only the pronounced P2 activities (P2I, P2II, P2III) were analyzed further. All other ERP components were evaluated within the interval of the first clapping sequence, that is, in the first 1000 ms.

**Figure 5:**
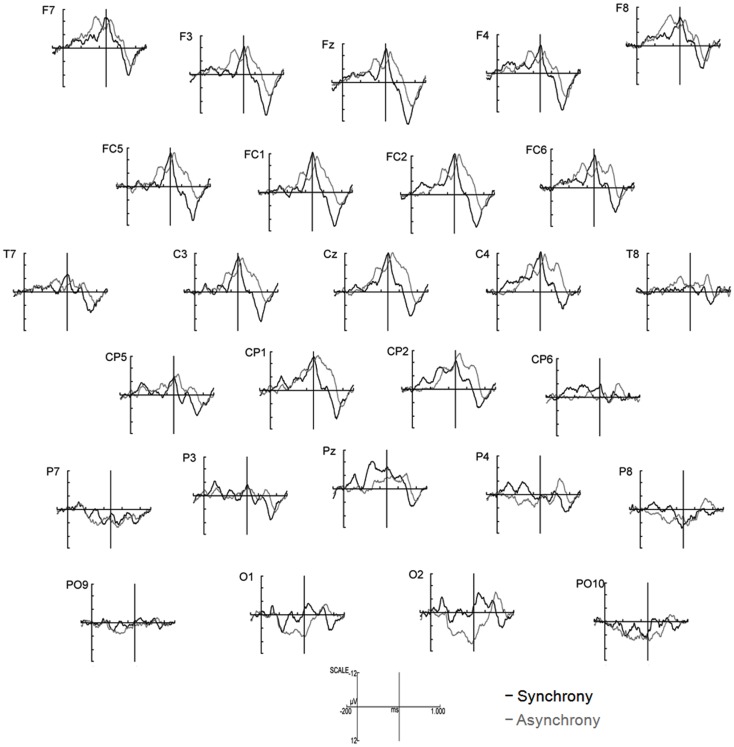
**ERPs elicited by synchronous (black line) and asynchronous (gray lines) stimuli in the first 1000 ms of stimulus presentation (*n* = 15)**. Note that the auditory event occurred at 474 ms in both experimental conditions (indicated by solid vertical lines).

**Figure 6:**
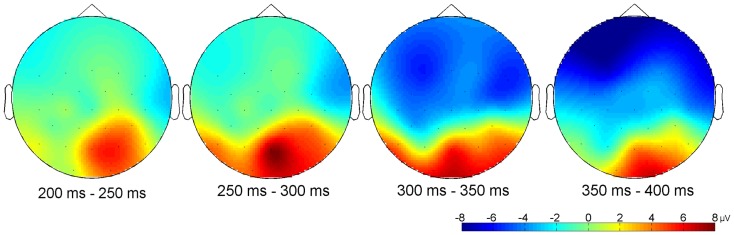
**Differential topographical map (asynchronous–synchronous trials) between 200 and 400 ms after stimulus onset (*n* = 15)**. Activity differences between the two stimuli emerged between 200 and 400 ms at posterior sites and between 300 and 400 ms at anterior electrodes.

Other relevant ERP components were identified at the beginning of the EEG epochs, indicating significant aspects of audiovisual interaction at the beginning of the rhythmic stimulus. The negative component Nc (interval: 450–650 ms after video onset) was elicited primarily at anterior sites. ERP curves and topographic maps (Figures 4–6) suggest that activity differences between experimental conditions emerged between 200 and 400 ms at posterior electrodes with asynchronous stimuli eliciting more positive deflections. In the time interval of the visual Pb component between 300 and 400 ms after video onset, ERP differences were observed at anterior electrodes with a polarity reversal in asynchronous stimuli.

Separate repeated-measures analyses of variance (ANOVA) were performed for the following dependent variables: peak amplitudes and peak latencies of N1, P2I, P2II, P2III, and Nc and mean amplitudes of Pb and posterior activity (200–400 ms). For all analyses, the significance level of α = 0.05 was Bonferroni-adjusted to control for multiple comparisons. Visual inspection suggested differential ERP activity between anterior and posterior sites (Figures 5 and 6). Therefore, topographic variability of the ERP components was tested first which resulted in the definition of regions of interest (ROI). This was done to prevent stimulus effects from remaining undetected in the less focal activity patterns of the infant ERP data. In addition, comparable ROIs related to similar experimental findings are still missing in the infancy literature. After the topographic analysis, specific effects of synchronous versus asynchronous stimuli were examined in the ROI electrodes while taking into account further possible lateralization effects.

#### Topography

Analyses of variances were performed including the within-subjects factors Stimulus (synchronous versus asynchronous) and Region with the following regions defined by electrode lines from anterior to posterior: frontal (F7, F3, Fz, F4, F8), fronto-central (FC5, FC1, FC2, FC6), central/temporal (T7, C3, Cz, C4, T8), centro-parietal (CP5, CP1, CP2, CP6), parietal (P7, P3, Pz, P4, P8), and parieto-occipital (PO9, O1, O2, PO10). Analyses revealed a main effect of Region on Nc amplitude, *F*(5, 70) = 7.81, *p* < 0.001, η^2^ = 0.36, mean posterior activity (200–400 ms), *F*(5, 70) = 6.31, *p* < 0.001, η^2^ = 0.31, and mean Pb activity, *F*(5, 70) = 5.50, *p* < 0.001, η^2^ = 0.28. More specifically, Nc amplitude was more negative in anterior than in posterior electrodes, whereas mean amplitude values between 200 and 400 ms and mean Pb amplitudes were on average negative at frontal, fronto-central, central/temporal, and centro-parietal electrodes but positive at parietal and parieto-occipital sites. The main effects of Region were qualified by reliable Region × Stimulus interactions on mean posterior activity (200–400 ms), *F*(5, 70) = 4.92, *p* = 0.001, η^2^ = 0.26, and mean Pb activity, *F*(5, 70) = 5.68, *p* < 0.001, η^2^ = 0.29. Accordingly, asynchronous stimuli elicited more negative voltages than synchronous stimuli in anterior electrodes but more positive amplitudes in parietal and parieto-occipital electrodes.

Furthermore, Region had a reliable main effect on latency of P2I, *F*(5, 70) = 17.35, *p* < 0.001, η^2^ = 0.55, P2II, *F*(5, 70) = 25.59, *p* < 0.001, η^2^ = 0.65, P2III, *F*(5, 70) = 14.68, *p* < 0.001, η^2^ = 0.51, and Nc, *F*(5, 70) = 5.46, *p* < 0.001, η^2^ = 0.28. In all four ERP components, latencies increased continuously from anterior to posterior. A reliable Region × Stimulus interaction was revealed for P2I latency, *F*(5, 70) = 3.82, *p* = 0.004, η^2^ = 0.21, and P2II latency, *F*(5, 70) = 2.57, *p* = 0.034, η^2^ = 0.16, with differential response latencies between synchronous and asynchronous stimuli at frontal, fronto-central, and central/temporal sites.

#### ROI analyses

Based on these statistical analyses and the topographic patterns (Figures 5 and 6), further analyses of specific Stimulus effects on N1, the P2s, and Nc as well as mean Pb activity were performed at frontal, fronto-central and central/temporal electrodes, whereas analyses of mean posterior activity (200–400 ms) were performed at parietal and occipital electrodes (for descriptive statistics of the Stimulus effect, see Table 1). To assess possible variations in lateralization, the within-subject factor Hemisphere was included in the ANOVAs with the following ROI: left-hemisphere electrodes (anterior: F7, F3, FC5, T7, C3; posterior: P7, P3, PO9), midline electrodes (anterior: Fz, FC1, FC2, Cz; posterior: Pz, O1, O2), and right-hemisphere electrodes (anterior: F4, F8, FC6, C4, T8; posterior: P4, P8, PO10).

**Table 1:** **Summary of descriptives of ERP components analyzed in synchronous and asynchronous trials in the specific regions of interest**.

			Mean	SD	Min	Max
N1	Amplitude (μV)	Synchrony	−3.6	9.4	−17.4	14.7
		Asynchrony	−7.6	8.8	−24.2	3.7
	Latency (ms)	Synchrony	630.5	47.7	563.9	723.4
		Asynchrony	694.9**	35.0	630.2	746.7
P2I	Amplitude (μV)	Synchrony	13.0	11.1	−4.2	32.2
		Asynchrony	12.6	8.5	0.2	30.1
	Latency (ms)	Synchrony	780.4	102.5	593.8	952.5
		Asynchrony	823.2^+^	110.4	643.0	1028.8
P2II	Amplitude (μV)	Synchrony	8.8	8.3	−2.0	31.3
		Asynchrony	11.2^++^	10.7	−2.0	44.0
	Latency (ms)	Synchrony	1755.4	116.1	1579.8	2000.4
		Asynchrony	1820.8**	83.4	1662.0	2029.4
P2III	Amplitude (μV)	Synchrony	12.8	7.1	3.1	31.6
		Asynchrony	12.1	9.5	−0.28	36.7
	Latency (ms)	Synchrony	2766.4	113.0	2601.3	3006.9
		Asynchrony	2820.8*	94.5	2647.2	3039.5
Nc	Amplitude (μV)	Synchrony	−12.3	8.2	−26.6	6.5
		Asynchrony	−12.2	9.2	−25.1	8.4
	Latency (ms)	Synchrony	526.8	69.9	449.8	664.8
		Asynchrony	601.4**	71.2	500.5	709.1
Pb	Mean amplitude (μV)	Synchrony	−1.3	9.0	−19.5	12.7
		Asynchrony	−5.2*	7.9	−18.6	14.6
Posterior activity (200–400 ms)	Mean amplitude (μV)	Synchrony	0.6	5.1	−10.5	11.5
		Asynchrony	3.5*	4.7	−6.1	9.9

*Descriptives are collapsed across the factor Hemisphere; analysis of N1, P2I, P2II, P2III, Nc, and Pb in anterior (frontal, fronto-central, central/temporal) electrodes and posterior activity (200–400 ms) in posterior (parietal, occipital) electrodes. *Min* and *Max* are reported here to document high inter-individual variability in infant EEG data*.

*Asynchrony* difference from *Synchrony*: *p < 0.05. **p < 0.01. ^+^*p* < 0.05 in left electrodes. ^++^*p* < 0.01 in midline electrodes.

#### N1

Peak amplitude was more negative in asynchronous than in synchronous stimuli but this difference did not reach statistical significance. In contrast, N1 latency was reliably shorter in synchronous than in asynchronous trials, *F*(1, 14) = 53.50, *p* < 0.001, η^2^ = 0.79.

#### P2I

Analyses revealed that P2I amplitude was more positive in synchronous than in asynchronous trials but – similar to N1 amplitude – this difference did not reach statistical significance. P2I latency to synchronous stimuli was marginally shorter than to asynchronous stimuli, *F*(1, 14) = 3.42, *p* = 0.085, η^2^ = 0.20. However, follow-up analyses for Hemisphere indicated that the difference between experimental conditions was significant at anterior left- and marginal at right-hemisphere electrodes [left: *F*(1, 14) = 5.38, *p* = 0.036, η^2^ = 0.28; right: *F*(1, 14) = 3.68, *p* = 0.076, η^2^ = 0.21].

#### P2II

In contrast to P2I, peak amplitude was affected in P2II. Voltage was reliably higher in asynchronous than in synchronous trials in anterior midline electrodes, as indicated by a significant Hemisphere × Stimulus interaction, *F*(2, 28) = 6.19, *p* = 0.006, η^2^ = 0.31. Furthermore, Stimulus had a significant effect on P2II latency, *F*(1, 14) = 9.21, *p* = 0.009, η^2^ = 0.40, with shorter latencies in synchronous than in asynchronous stimuli.

#### P2III

No significant effects were found for P2III amplitude. Again, a significant main effect of Stimulus on P2III latency, *F*(1, 14) = 5.70, *p* = 0.032, η^2^ = 0.29, indicated shorter latencies for synchronous than for asynchronous stimuli. Topographic variations in P2III latency were reflected in a reliable Hemisphere effect, *F*(2, 28) = 3.67, *p* = 0.038, η^2^ = 0.21. Accordingly, P2III activity peaks occurred earlier on average in anterior left-hemisphere (*M* = 2778.5 ms) than in anterior midline electrodes (*M* = 2803.8 ms).

#### Nc

Hemisphere had a significant main effect on Nc amplitude, *F*(2, 28) = 4.62, *p* = 0.018, η^2^ = 0.25, with smaller (less negative) peak amplitudes in anterior left-hemisphere (*M* = −10.2 μV) than anterior midline (*M* = −13.8 μV) and anterior right-hemisphere (*M* = −12.6 μV) electrodes. As with the P2s, Nc latency was reliably shifted between synchronous and asynchronous trials, *F*(1, 14) = 12.30, *p* = 0.003, η^2^ = 0.47.

#### Comparison of N1, P2I, P2II, P2III, and Nc latency

**Figure 7:**
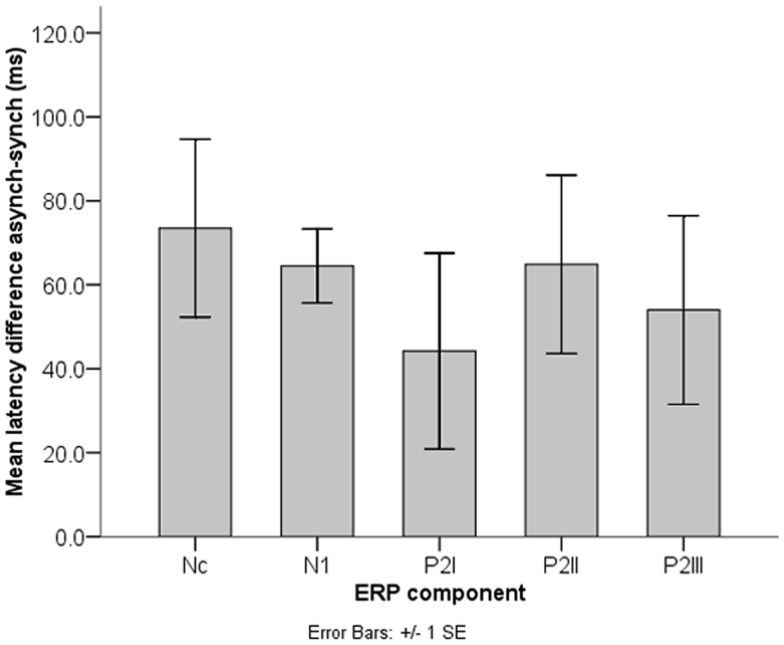
**Nc, N1, and P2 latency shifts (*n* = 15)**. Latency differences between synchronous and asynchronous stimuli did not differ significantly between the ERP components and were always significantly smaller than the AV asynchrony of 400 ms.

The significant peak latency differences between synchrony and asynchrony raise the question whether these temporal shifts vary within the trial. Figure 7 illustrates mean latency differences in Nc, N1, P2I, P2II, and P2III. A repeated-measures ANOVA including the within-subject factor Component (P2I versus P2II versus P2III) revealed no differences in the latency shifts between the individual P2 components, *F* (2, 28) = 0.46, *p* = 0.630. Furthermore, including N1 and Nc in the same analysis yielded comparable results, *F*(4, 56) = 0.50, *p* = 0.737. Thus, a constant difference in temporal alignment can be assumed. At the same time, these latency shifts between synchronous and asynchronous stimuli were significantly different from the AV delay of 400 ms in all five ERP components, as indicated by one-sample *t* tests [N1: *t*(14) = 38.05, *p* < 0.001; P2I: *t*(14) = 15.24, *p* < 0.001; P2II: *t*(14) = 15.76, *p* < 0.001; P2III: *t*(14) = 15.40, *p* < 0.001; Nc: *t*(14) = 15.41, *p* < 0.001].

#### Mean Pb activity

Asynchronous stimuli elicited reliably more negative mean amplitudes than did synchronous stimuli, *F*(1, 14) = 5.85, *p* = 0.030, η^2^ = 0.30 (Figures 5 and 6). The Hemisphere × Stimulus interaction was not significant, *F*(2, 28) = 2.21, *p* = 0.128.

#### Mean posterior activity (200–400 ms)

Stimulus had a significant main effect on mean amplitude, *F*(1, 14) = 6.13, *p* = 0.027, η^2^ = 0.30, with more positive amplitudes in asynchronous than in synchronous trials. Visual inspection of posterior activity (200–400 ms) suggests more right-lateralized contributions to this effect (Figure 6). However, Hemisphere neither had a main effect nor interacted with Stimulus.

#### Behavioral covariate

The ADS, as an indicator for the magnitude of recovery of interest to AV400 after habituation to the synchronous stimulus, was entered into separate ANOVAs as a covariate. Dependent variables were the same as in the ROI analyses. There was a reliable Hemisphere × ADS interaction on mean posterior activity (200–400 ms), *F*(2, 26) = 5.23, *p* = 0.012, η^2^ = 0.29. That is, the higher the ADS, the more positive the mean activity between 200 and 400 ms was at posterior right electrodes [simple effect of ADS at posterior right electrodes: *F*(1, 13) = 6.46, *p* = 0.025, η^2^ = 0.33]. This general lateralized activity pattern was independent of stimulus type.

## Discussion

The results from the habituation-test paradigm of the present study confirm earlier work (Lewkowicz, 1996) by showing that, at the group level, infants in the first year of life are able to detect audiovisual asynchronies of 400 ms in non-speech stimuli. The degree of recovery of interest varied between infants. Interestingly, the magnitude of the behavioral ADS was correlated to neural activity as early as 200–400 ms at right posterior sites. In other words, the longer children gazed to the asynchrony, the larger a general neural responsiveness to this type of stimulus was, independent of temporal synchrony relations. With respect to the general infant population, however, one has to keep in mind that the result very likely overestimates the correlation as a result of the exclusion of children with fewer or no signs of dishabituation.

As predicted, ERP differences between synchronous and asynchronous events were evident in the auditory components N1 and P2. In adults, N1 and P2 amplitudes were shown to decrease in response to multisensory as compared to unisensory stimuli when both modalities were presented in synchrony (e.g., van Wassenhove et al., 2005; Stekelenburg and Vroomen, 2007; Besle et al., 2009) but not in asynchrony (Pilling, 2009). In the present study, N1 amplitudes tended to be less negative and P2II amplitudes were less positive in response to synchrony than to asynchrony. The amplitude reductions in synchronous audiovisual events resemble those described in adult research as reflecting neural interactions between the two modalities (Besle et al., 2009; Pilling, 2009). However, in the infant sample, N1 and P2 amplitude modulations were not statistically systematic (differences between synchronous and asynchronous stimuli were present in P2II but not significant in N1, P2I, and P2III; cf. Hyde et al., 2011). One could assume that this sort of pattern develops with increasing age. In addition, high intra- and inter-individual variability in infant ERPs or sample size may affect the results. Moreover, amplitude reductions may be of greater significance in bimodal interactions versus unimodal presentations than in the manipulation of temporal synchrony relations.

In contrast, the most striking and statistically robust result here is the constant temporal delay of auditory ERP peaks between synchronous and asynchronous stimuli (Figure 7), with on average significantly longer latencies in the asynchronous condition in N1 and the P2s. This finding is important given that the auditory events were presented at the same point in time in both conditions (sound onsets: 474, 1474, and 2474 ms). Apparently, neural activity elicited by the auditory events was mostly delayed toward the corresponding visual activity. This temporal delay was maintained throughout the trial (Figure 7). As the behavioral data show, perceptual fusion is very unlikely (cf. Miller and D’Esposito, 2005; Stevenson et al., 2011). One could argue that these significant latency delays in the auditory ERP components may be an indicator for the emergence of an asynchronous percept on the behavioral level. Further, the temporal shift of auditory brain activity could reflect an attempt of the infant brain to operate against asynchrony (Hyde et al., 2011), despite the clear temporal disparity of vision and audition. At the same time, latency differences between synchronous and asynchronous stimuli in N1, P2I, P2II, and P2III were significantly smaller than the delay of 400 ms in the visual stream, thus suggesting a temporal interaction between the two modalities. Apparently 6 months or less of multisensory experience with synchronous events and/or exposure to the hand-clapping stimuli in the habituation task were sufficient to develop these processes.

A synchrony detection mechanism for binding across sensory modalities has been proposed in earlier research in adults (e.g., Navarra et al., 2005). Accordingly, temporally misaligned auditory and visual events are drawn into approximate temporal register, whereby the brain pulls auditory signals into temporal alignment with the corresponding visual stream (Vatakis et al., 2007; Navarra et al., 2009). The results of our experiment confirm these suggestions and show that similar mechanisms might operate in 6-month-old infants, at least in the present paradigm (but, see also Fort et al., 2002; Lewkowicz, 2010). Here, although the visual information lagged behind the corresponding auditory stream, infants also saw the hand movements of the asynchronous stimulus before sound onset, which might have initiated the delay in neural activity.

Similar latency effects in the adult ERP were reported to vary with predictability of auditory signals. That is, the temporal facilitation in N1 and P2 increased with higher salience of visual inputs (van Wassenhove et al., 2005). In our hand-clapping stimuli, predictability was high, as the sound onset could be predicted from the hand movements. Thus the latency shifts of the auditory N1 and P2 in the infant sample cannot be considered independent of prior visual motion. Indeed, results show that a significant latency shift was already revealed in the preceding Nc component. Again, longer latencies were observed in the asynchronous condition. Nc modulations have typically been associated with attentional mechanisms in infant visual recognition and memory (e.g., de Haan and Nelson, 1997; Ackles, 2008; Kopp and Lindenberger, 2011, 2012). Hence, the present Nc latency differences might imply an attentional shift in time between synchronous and asynchronous stimuli. The temporal course of the ERP pattern makes it very unlikely that Nc activity is a direct response to the physical sound onset. Rather the latency shift might be a result of the anticipated sound onset in each of the two conditions. There was on average no significant difference in the magnitude of the latency shifts between Nc, N1, and the P2s, which indicates that temporal activity relations between vision and audition were maintained throughout the rhythmic stimulus, once they had been established after video onset. The fact that these relations were present even before the auditory onset suggests prediction or anticipation processes in the infant brain that might trigger neural activity in the remaining stimulus epoch.

In fact, the data of this experiment show that differential activation between synchrony and asynchrony perception began as early as 200 ms after video onset (approximately 270 ms before sound onset). It propagated from posterior to anterior sites (Figure 6). Audiovisual asynchrony effectively elicited a polarity reversal of the Pb component in the anterior ERP. The functional significance of Pb in infant visual recognition paradigms is still under investigation. It has been related to mechanisms of stimulus expectancy, particularly to the probability of occurrence of an event (Karrer and Monti, 1995; Kopp and Lindenberger, 2011, 2012). Here, one could argue that the visual recognition process might be altered and that Pb modulations indicate differences in the infants’ expectation of the hand-clapping movement. However, the visual stream in the asynchrony condition was not *per se* an odd or deviant stimulus. Instead, it was the same biologically possible motion as in the synchrony condition, and it was also presented with the same frequency during the experimental session. Hence, Pb activity differences between the two conditions suggest that the visual motion was perceived relative to the auditory event that occurred later in the trial and that could be expected at the same time point in both conditions. In other words, the 6-month-old infants of the present sample might have predicted audiovisual temporal synchrony relations of the current event (see also Petrini et al., 2009b). Future studies could include other visual control conditions to explore these dynamics further, provided that infants’ attention allows for a longer test session. It is known from infant ERP research that, in visual recognition paradigms, unisensory stimuli with comparable onset time and duration elicit Pb activity with comparable polarity and latency and typically elicit amplitude modulations due to stimulus characteristics (e.g., Karrer and Monti, 1995; Hill Karrer et al., 1998; Webb et al., 2005). Hence one would assume that the mere temporal delay of the content of the visual stream should elicit similar Pb amplitude modulations in unisensory visual stimuli, but not a polarity reversal followed by Nc latency shifts. In other words, it is fair to conclude that the present ERP pattern of the asynchronous condition before sound onset (474 ms) is likely to reflect aspects of stimulus processing that are related to the multisensory nature of the event.

In contrast to earlier ERP findings on multisensory perception in 5-month-old infants (Hyde et al., 2011), the present data did not show consistent P2 amplitude modulations, and differences were also found in other ERP components. However, as noted in the introduction, the present paradigm investigating the perception of temporal synchrony relations was not directly comparable to the earlier work. With a static visual input in Hyde et al. (2011), Nc peak latency differences corresponding to the magnitude of the stimulus asynchrony were found. With a dynamic but modified visual input, no peak latency differences were observed. In contrast, using dynamic audiovisual presentations with temporally shifted visual content in our study, Nc and P2 latencies showed temporal interaction patterns, indicating dynamic perceptual binding mechanisms.

Next to the stimulus presentation, several other variables may potentially contribute to our results. First, type and complexity of the stimuli could be a source of variability. For example, it is known that perceptual binding in the temporal range of audiovisual integration is different for speech and non-speech events, both in adults (Dixon and Spitz, 1980) and in infants (Lewkowicz, 2010). Here, we used social non-speech stimuli. However, ERP modulations due to audiovisual interactions in adults were found to be similar between speech- and non-speech stimuli (Stekelenburg and Vroomen, 2007; Vroomen and Stekelenburg, 2009). Also, as noted above, anticipatory visual motion affected auditory N1 and P2 activity modulations in adults (Stekelenburg and Vroomen, 2007; Vroomen and Stekelenburg, 2009). Preceding visual movement reduced the uncertainty about an auditory onset (Besle et al., 2009), and with increased saliency of the visual input, predictability of an auditory signal also increased (van Wassenhove et al., 2005). It would certainly be interesting to investigate how the infant brain responds to stimuli without such anticipatory visual motion (cf. Stekelenburg and Vroomen, 2007). Furthermore, taking the infants’ relatively short multisensory experience into account, the question arises how neural responses to temporal synchrony relations would look with completely novel stimuli. In other words, the present results are likely to reflect – at least to some extent – the influence of cumulative experience with the stimuli during the habituation and EEG paradigm. In addition to the present experimental manipulation, further control conditions could be helpful to elucidate the dynamics of multisensory processing. However, in infant samples, multiple conditions and repeated stimulus presentation may produce unacceptably high attrition rates and may have an effect on the ERP findings and their generalizability (for further discussion, see Snyder et al., 2002; Stets et al., 2012). Finally, in order to understand developmental changes of multisensory perception, future studies should investigate EEG activity in younger and older children and in adults. Taking general neural maturation processes into account, the present paradigm could help identify indicators of audiovisual temporal perception processes.

In the present study, visual and auditory ERP activity overlapped. Hence, when aiming for the separation of audition and vision and the application of an additive model (cf. Foxe and Molholm, 2009), a clear dissociation between the ERP components of each individual modality may be difficult (e.g., see discussion in Talsma et al., 2009). In addition, a comparison of ERP activity time-locked to the visual clapping would be complicated because of the lack of comparable stimulus onsets, ERP component activity relative to stimulus onset, and baseline activity (see Figure 4). The two experimental conditions in our study differed only in the temporal content of the visual input while visual and auditory onsets and sequence lengths did not vary. As the time course of the overlap of unisensory modalities is highly similar between synchronous and asynchronous stimuli, a direct comparison of ERP peaks between conditions seems valid. It is important to note that differences between the conditions already arose before the onset of auditory input. Hence, an overlap of visual and auditory ERP components alone cannot explain the neural activity patterns without taking into account temporal synchrony relations. Furthermore, from the perspective of ecological validity, one could argue that a purely unisensory input is rather unusual outside the laboratory. Here, the aim was to study multisensory stimulus processing as it could occur in everyday situations. For example, in a video internet call, the visual stream may be delayed with respect to the auditory signal due to technical constraints, and simultaneity perception might be difficult or impossible. We think that the brain adapts perfectly to naturalistic stimuli, and developmental mechanisms should be investigated in such contexts. This study shows that the perceptual system does not need years of multisensory experience to develop a unity assumption (Welch and Warren, 1980) and to anticipate temporal synchrony relations between vision and audition.

To conclude, we confirmed infants’ sensitivity to temporal synchrony relations in ecologically valid, non-speech stimuli, and demonstrated temporal interactions between vision and audition in the infant brain toward perceptual fusion although the magnitude of the asynchrony did not allow for simultaneity perception. The present results suggest anticipatory mechanisms and predictive capacities as to audiovisual temporal synchrony relations in infants as young as 6 months of age.

## Conflict of Interest Statement

The authors declare that the research was conducted in the absence of any commercial or financial relationships that could be construed as a potential conflict of interest.

## Footnotes

^1^Due to high inter-individual variability in infant gazing behavior, significant response recovery can often be observed only in group-level data in which effects of different presentation orders are counterbalanced. Here, we chose an individual data approach including the same order of test trials for each infant. As this presentation mode could result in over- or underestimation of response recovery, we included gaze at the last habituation trial into the analysis.
